# Improvement of the sensitivity of circulating tumor DNA-based liquid biopsy: current approaches and future perspectives

**DOI:** 10.37349/etat.2025.1002333

**Published:** 2025-08-08

**Authors:** Ekaterina S. Kuligina, Grigoriy A. Yanus, Evgeny N. Imyanitov

**Affiliations:** University of Campania “Luigi Vanvitelli”, Italy; ^1^Department of Tumor Growth Biology, N.N. Petrov Institute of Oncology, 197758 St.-Petersburg, Russia; ^2^Department of Medical Genetics, St.-Petersburg State Pediatric Medical University, 194100 St.-Petersburg, Russia

**Keywords:** Liquid biopsy, cancer therapy, circulating tumor DNA, next-generation sequencing, analytical performance, reproducibility, circulating tumor DNA assays

## Abstract

Liquid biopsy (LB) is a complex of procedures aimed at the detection of tumor-derived fragments (nucleic acids, proteins, cells, etc.) persisting in the blood or other body fluids. It can be utilized for early cancer diagnosis, analysis of biomarkers of tumor drug sensitivity and prognosis, monitoring of minimal residual disease (MRD), etc. Circulating tumor DNA (ctDNA) is an accessible and reliable LB analyte as it may contain tumor-specific mutations and is amenable to efficient detection by next-generation sequencing (NGS) or droplet digital PCR (ddPCR). High level of ctDNA is typically associated with increased tumor burden and poor prognosis, whereas treatment-related ctDNA clearance increases the probability of a favorable disease outcome. Major efforts have been invested in enhancing the analytical performance of ctDNA detection. Stimulation of apoptosis of tumor cells by irradiation of cancer lumps has been shown to result in a transient but modest increase in ctDNA concentration. There are several sophisticated modifications of ultra-deep NGS protocols, which discriminate between “true” low-copy mutation-specific signals and sequencing artifacts. Slowing physiological ctDNA decay by interfering with liver macrophages and circulating nucleases has shown promise in animal experiments. Reproducibility of ctDNA-based LB assays remains insufficient for samples with ultra-low content of ctDNA; hence, interlaboratory harmonization of ctDNA testing procedures is of paramount importance.

## Introduction

Solid tumors often shed their fragments into the bloodstream and other body fluids. These include live cancer cells or even small multicellular tumor pieces, some tissue-specific proteins, circulating tumor DNA (ctDNA), microRNAs (miRNAs), etc. Detection of these tumor-specific traces is often called liquid biopsy (LB), indicating its potential interchangeability with the invasive extraction of the tumor material. First LB methods were developed several decades ago and relied on the detection of tumor-derived proteins, such as carcinoembryonic antigen (CEA) [1, 2], prostate-specific antigen (PSA) [3, 4], ovarian cancer marker CA-125 [5, 6], etc. These techniques are currently routinely utilized during the diagnostic procedures, the assessment of the efficacy of surgical and therapeutic interventions, and the monitoring of the disease relapse [7, 8]. Furthermore, despite being rather tissue-specific than cancer-specific, protein markers are included in some screening programs [9].

Molecular genetic studies revealed that tumors shed into body fluids not only proteins but also nuclear DNA. This DNA probably originates from cancer cells undergoing apoptosis [10–12]. The pattern of driver and passenger mutations in ctDNA is usually identical or highly similar to the one observed in the corresponding primary tumor [13–16]. Mutated DNAs are generally more cancer-specific than other biomarkers. Furthermore, current laboratory techniques allow for the detection of single mutated DNA copies in the presence of a huge excess of normal DNA; therefore, ctDNA analysis has become a widely utilized LB option [17–21]. In addition, the investigation of ctDNA may serve not only as a proof of the mere presence of cancer disease but provide knowledge on molecular targets present in the tumor tissue [22–25]. Elevated concentration of ctDNA in treatment-naïve cancer patients is known to be associated with poor prognosis [26–28]. Changes in plasma ctDNA concentrations closely reflect the extent of tumor burden during the natural history of cancer disease and its response to the treatment [29–31]. Notably, ctDNA is more frequently detected in tumors with vascular invasion [32]. Collectively, these findings underscore the prognostic utility of ctDNA across a range of tumor types [33].

The content of ctDNA in the bloodstream of cancer patients is vanishingly low, being less than 1–100 copies per 1 mL of plasma [34–36]. In early-stage tumors, only a very tiny portion of cells undergo apoptosis and shed DNA [37, 38]. Furthermore, ctDNA is rapidly eliminated from the bloodstream by liver macrophages and soluble nucleases [39–42]. Virtually all ctDNA assays are performed at the limit of their technical possibilities.

**Figure 1:**
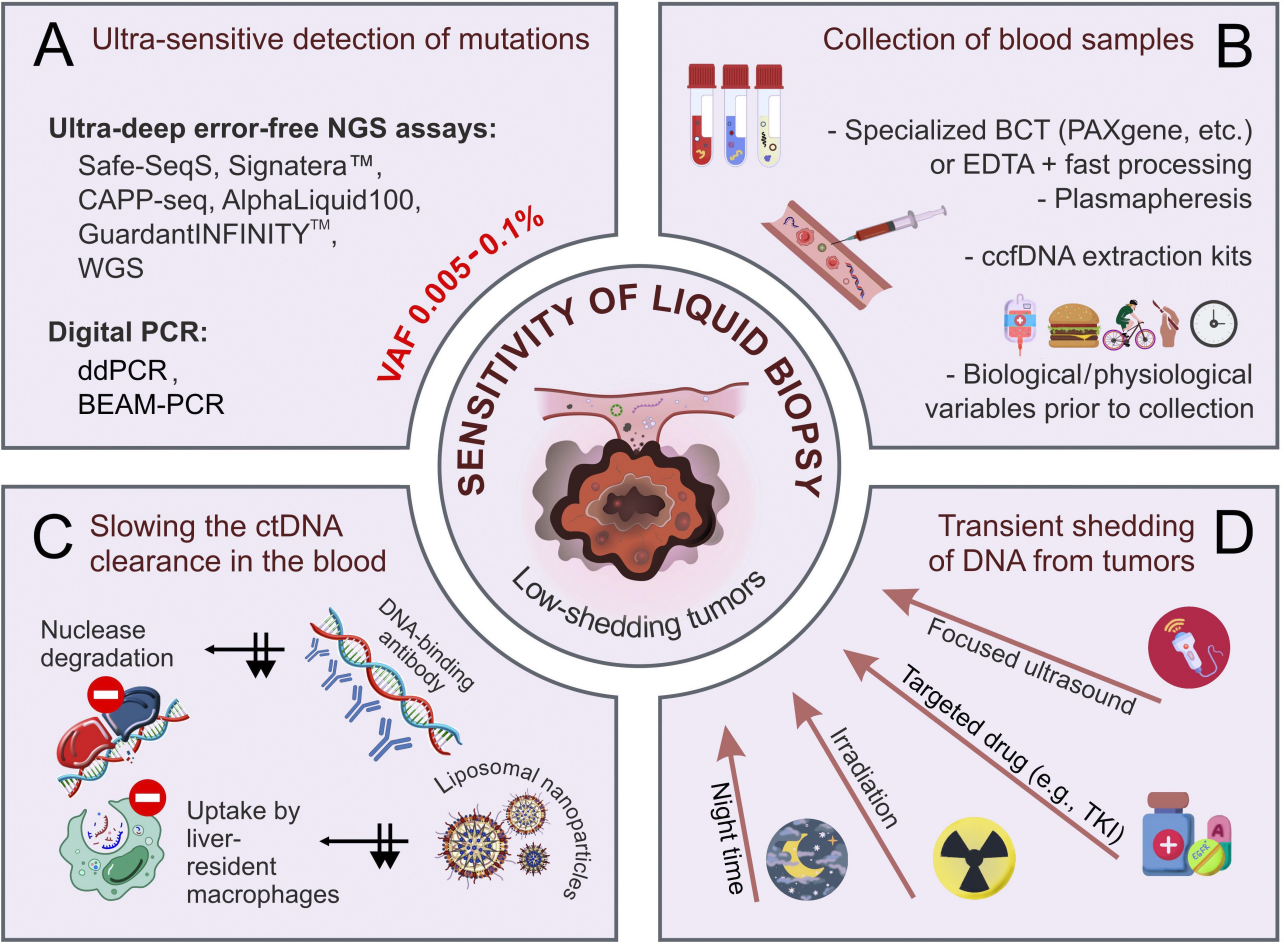
**Improvement of the analytical performance of ctDNA-based liquid biopsy.** (**A**) Ultra-sensitive methods of detection of mutated DNA sequences; (**B**) pre-analytical factors affecting the quality and yield of ctDNA; (**C**) slowing the ctDNA clearance in the blood (black arrows indicate the inhibition of two routes of ctDNA clearance); (**D**) transient shedding of DNA from tumors. BCT: blood collection tubes; BEAM: beads, emulsions, amplification, and magnetics; ccfDNA: circulating cell-free DNA; ctDNA: circulating tumor DNA; ddPCR: droplet digital PCR; EDTA: ethylenediaminetetraacetic acid; NGS: next-generation sequencing; TKI: tyrosine kinase inhibitors; VAF: variant allele frequency; WGS: whole-genome sequencing. Some images were adapted using free resources from Flaticon.com

There are several avenues for the improvement of the performance of ctDNA testing (Figure 1). First, an “ideal” ctDNA assay must be able to detect, say, 1 mutated DNA molecule per 10–25 mL of blood (4–10 mL of plasma) [43–45]. Still, this may not be enough for clinical purposes, as plasma obtained from patients with small tumor burden (below 1–10 grams) often contains an order of magnitude less amount of ctDNA [46–48]. There are studies suggesting that ctDNA release may be stimulated by a variety of factors such as irradiation, ultrasound, mechanical stress, etc. [49–52]. Alternatively, interference with in vivo physiological and pathological processes may influence the proportion of tumor-derived DNA in total circulating cell-free DNA (ccfDNA) [53, 54]. This article provides an overview of practical approaches aimed at increasing the sensitivity of ctDNA detection.

## Collection of biospecimens

Normally, blood contains up to several thousand copies of wild-type extracellular DNA per 1 mL of plasma [55, 56]. This background compromises the detection of tumor-derived mutated DNA. The concentration of circulating “normal” DNA is generally higher in the elderly than in young people [57, 58]. Furthermore, some inflammatory or autoimmune diseases, physical exercise, trauma, etc., may increase the overall content of ccfDNA [59–66]. Tumor-derived DNA usually constitutes no more than 0.025–2.5% of circulating fluid DNA [34, 36]. This proportion depends on the biological and genetic properties of the tumor, overall amount of malignant cells within the body, and various treatment-related factors [20, 67–73]. The release of DNA by blood cells is a major factor confounding ctDNA analysis [35]. At present, all protocols for ctDNA extraction require careful separation of plasma from blood cells and cellular debris, which is achieved by two rounds of centrifugation and manual saving of ctDNA-containing samples. Conventional ethylenediaminetetraacetic acid (EDTA)-containing tubes require almost immediate processing of the blood, with the waiting time not exceeding 2–6 hours at 4°C [74, 75]. There are several blood collection tubes (BCT), e.g., cfDNA (Streck), PAXgene Blood ccfDNA (Qiagen), cfDNA/cfRNA Preservative (Norgene), ImproGene cfDNA (Improve Medical), cfDNA (Roche), etc., which contain some stabilizers of the integrity of nucleated blood cells, thus preventing the release of normal genomic DNA and minimizing the hemolysis. They all allow for the storage and transportation of blood samples for up to 7 days at room temperature [53, 76, 77]. Significant efforts have been invested in head-to-head comparisons of the tubes obtained from different manufacturers, with some minor differences revealed [78–80]. Although these tubes are convenient for the ctDNA analysis, they are not always compatible with the multianalyte LB, as they may not permit simultaneous analysis of circulating tumor cells (CTCs), protein markers, extracellular vesicles (EVs), etc. Consequently, many investigators opt for conventional EDTA tubes despite the requirement for immediate blood processing [81–85] (Table 1).

**Table 1:** Critical pre-analytical features of ctDNA-based LB workflow

**Stage**	**Recommendation**	**Notes**	**References**
Blood collection:			
Procedure	Use of butterfly needles	Avoid excessively thin needles and prolonged tourniquet use.	[53, 86–88]
	Plasmapheresis/leukapheresis	Apply microfluidic enrichment and FACS.	[89, 90]
Sample volume	2 × 10 mL of blood (for single-analyte LB)	Screening, MRD detection, WGS, and testing of multiple analytes may necessitate larger plasma volumes.	[81, 91, 92]
BCT	EDTA tubes	Require fast processing (within 2–6 hours).	[74, 75, 85]
	BCT with cell stabilizing preservative agents: cfDNA (Streck), PAXgene Blood ccfDNA (Qiagen), cfDNA/cfRNA Preservative (Norgene), ImproGene cfDNA (Improve Medical), cfDNA (Roche)	Preserve the quality of ctDNA samples within 3–7 days at a temperature of 4–25°C.	[77, 78, 84]
Biological features	Control for physical activity, and physiological and pathological status prior to blood collection	Chronic or acute diseases (e.g., diabetes, endometriosis, obesity, kidney disease, hypertension, and inflammation) are associated with elevated ccfDNA content.	[61–63]
	Surgical trauma	Surgical trauma causes a transient increase in the level of ccfDNA, which persists for up to a few weeks after surgery.	[59, 93]
	Circadian dynamics	Increase of CTC and ctDNA content at night.	[94, 95]
Induction of transient ctDNA release from tumor before blood take	Irradiation	The spike of ctDNA concentration in 6–24 hours after the procedure.	[51, 96–99]
	Ultrasound	Sonobiopsy for brain tumors.	[50, 52, 100]
	Mechanical stress	Mammography for breast cancer; digital rectal examination for prostate cancer.	[49, 101, 102]
Transportation and handling	Use special BCT for long-distance transportation. EDTA tubes are good only for transportation within a hospital	Avoid high temperature, stirring, or violent vibration during transportation.	[78, 85, 103]
Plasma processing:			
Centrifugation	Double centrifugation: 1st step (slow centrifugal force: 380–3,000 *g* for 10 min at room temperature), 2nd step (12,000–20,000 *g* for 10 min at 4°C)	Single low-speed centrifugation is recommended for PEG-mediated enrichment.	[53, 85, 104, 105]
Cell-free plasma storage	At –80°C	10 years for mutation detection; 9 months for quantitative analysis.	[53]
Thawing of stored plasma	Slowly on ice	Freeze-thaw cycles must be minimized; it is recommended to store the plasma in small fractions.	[106, 107]
ctDNA extraction:			
Chemistry	Solid phase extraction: - Silica membrane columns: QIAamp Circulating Nucleic Acids Kit (Qiagen); Cobas ccfDNA Sample Preparation Kit; - Magnetic beads: QIAamp MinElute ccfDNA Mini Kit (Qiagen); Maxwell RSC LV ccfDNA Kit (Promega); MagNa Pure 24 Total NA Isolation Kit (Roche)	Silica membrane-based kits yield more ctDNA than methods utilizing magnetic beads.	[81, 106, 108–111]
	Liquid phase extraction	Utilize the standard phenol-chloroform extraction or specially designed phase-forming aqueous systems.	[112, 113]
	Microfluidic platforms	Cost-efficient approach allowing for rapid isolation, detection, and characterization of ctDNA.	[114–116]
	Addition of polymers to the blood sample (e.g., PEG)	Improves the quantity and purity of ctDNA; facilitates precipitation of extracellular vesicles, lipoproteins, and ribonucleoprotein complexes, thus providing the access to multianalyte assays.	[105, 117, 118]
Workflow	Moving toward standardized automatic methodologies and multianalyte extraction protocols	Reduce the hands-on time of the extraction.	[106, 119–121]

BCT: blood collection tubes; ccfDNA: circulating cell-free DNA; cfDNA: cell-free DNA; CTC: circulating tumor cell; ctDNA: circulating tumor DNA; EDTA: ethylenediaminetetraacetic acid; FACS: fluorescence-activated cell sorting; LB: liquid biopsy; MRD: minimal residual disease; PEG: polyethylene glycol; WGS: whole-genome sequencing

Plasma collection is a serious confounding factor in the real-world clinical setting, as it requires significant manual work and is poorly amenable to standardization. Ideally, an automated device near the blood-draw facility is needed to process the blood and plasma.

Several studies have highlighted a suitability of dried blood spots (DBS) collected via finger-prick microsampling for ctDNA analysis. This approach is compatible with the whole-genome sequencing (WGS) and allows the detection of somatic copy number aberrations, fragment length profiles, tumor-specific single-nucleotide mutations, etc., across various cancer types [122, 123]. The protocol for ctDNA isolation from DBS includes a bead-based size selection step that effectively distinguishes tumor-derived cfDNA from background genomic DNA. Considering that DNA and RNA remain stably preserved on cards for extended periods [124], the use of DBS presents a logistical advantage for practical utilization of ctDNA-based assays.

## ctDNA extraction

The overall amount of ctDNA in plasma samples is usually estimated in nanograms [35, 125]. Exhaustive isolation of these tiny quantities of DNA is a challenge, especially given that it is partially degraded (Table 1). Most laboratories utilize kits, which collect DNA on a solid phase. Silica membrane columns [e.g., QIAamp Circulating Nucleic Acids Kit (Qiagen), Cobas cfDNA Sample Preparation Kit (Roche), etc.] require significant manual efforts; however, some comparative studies demonstrated that they produce superior DNA yields [106, 108–110]. Protocols involving magnetic beads [e.g., QIAamp MinElute ccfDNA Mini Kit (Qiagen), Maxwell RSC LV ccfDNA Kit (Promega), MagNa Pure 24 Total NA Isolation Kit (Roche)] are more amenable to some automation, although their performance has been questioned because of lower ctDNA recovery [106].

While the majority of laboratories rely on the absorption of ctDNA on membranes or beads [81, 106, 108–111], some researchers prefer ethanol-based precipitation of nucleic acids from a liquid phase [112, 113, 126]. Purification of ctDNA may be achieved either by conventional phenol-chloroform extraction or by more sophisticated reagents containing polymers, salts, ionic liquids, and surfactant components. There are data suggesting that these methods offer even higher ctDNA recovery and purity compared to solid-phase systems [112, 113].

The addition of polyethylene glycol (PEG) to the plasma was shown to improve ctDNA isolation [105, 117, 118]. There are also some microfluidic platforms providing rapid and cost-efficient isolation of circulating DNA [114–116].

## Detection of mutations in ctDNA

There are two major avenues for the analysis of tumor-derived ctDNA [127–134]. Post-treatment follow-up often relies on a tumor-informed approach, in which the assay is designed in accordance with the pattern of mutations in the cancer tissue [127, 128]. This procedure may utilize both droplet digital PCR (ddPCR) and next-generation sequencing (NGS), and it is generally less prone to various artifacts [129, 130]. The tumor-agnostic approach is the only viable tool for early cancer detection because it relies on NGS analysis of a wide spectrum of commonly mutated cancer genes or sequencing of the entire genome [131–134]. It is essential to emphasize that tumors are not the only source of mutated DNA. Clonal hematopoiesis of indeterminate potential (CHIP) is also characterized by oncogenic mutations and is particularly common in elderly individuals [135, 136].

Earlier ctDNA studies used conventional real-time PCR for the detection of tumor-associated mutations in plasma samples [137, 138]. Real-time PCR has insufficient sensitivity and specificity for ctDNA analysis, and its use is discouraged.

ddPCR is the most appropriate method for tumor-informed ctDNA analysis, as it allows for reliable detection of mutated gene copies in the presence of at least 1,000-fold or even higher excess of normal DNA [20, 139–141]. NGS is about an order of magnitude less sensitive than ddPCR; however, this disadvantage is compensated by simultaneous analysis of multiple mutated sites [37, 43, 127, 128, 142–144].

Both ddPCR and NGS procedures involve DNA synthesis, which is prone to occasional errors [145]. This is a common drawback; therefore, the presence of a single mutation-specific signal in ddPCR or NGS run cannot be reliably interpreted in favor of mutation. Current NGS technologies utilize elegant modifications, which have been specifically developed for the detection of mosaic mutations, for example, Safe-SeqS [146], Signatera^™^ [127], RaDaR^™^ [147], Avenio (CAPP-seq) [148], Guardant360 [149], AlphaLiquid100 [23, 150], or some laboratory-developed tests [151].

**Figure 2:**
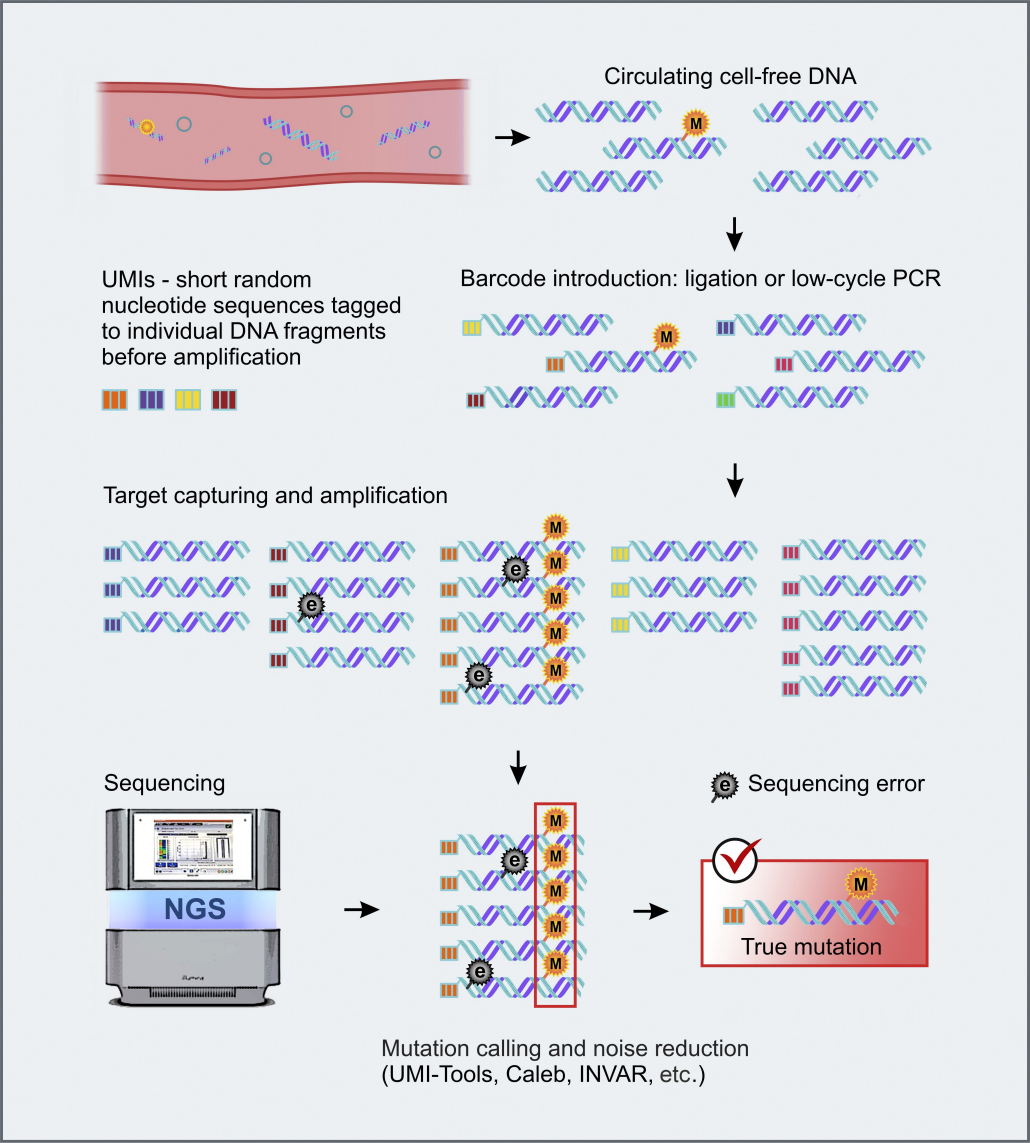
**UMI-barcoding strategy for background noise reduction.** e: sequencing error; M: true mutation. NGS: next-generation sequencing; UMIs: unique molecular identifiers. Some images were adapted using free resources from Flaticon.com

The performance of NGS-based low-copy ctDNA detection can be improved by the use of so-called unique molecular identifiers (UMIs) [152]. UMIs are random DNA sequences, which are added to individual ctDNA molecules before PCR amplification (Figure 2). Consequently, all PCR products originating from a given DNA molecule (i.e., having the same oligonucleotide identifier) can be recognized and subjected to an individual bioinformatics analysis. Sequence differences between the reads originating from the same template are interpreted as errors. These NGS modifications permit accurate identification of mutations in the presence of 10,000-fold excess of normal DNA [134, 153, 154]. Duplex sequencing employs random tagging of each individual DNA duplexes during library preparation, allowing the identification of all amplified DNA fragments originating from a single strand of the original DNA molecule [155]. The mutation is considered real only if both strands forming a UMI-labelled fragment reveal complementary alterations in a given nucleotide position [156].

Combining NGS with artificial intelligence (AI) and machine learning (ML) tools for mutation calling has been shown to reduce the background noise, thus enabling accurate detection of true ctDNA alterations at variant allele frequency (VAF) as low as 10^–5^ [157–160]. Some ML tools, e.g., Random Forest classifiers, K-nearest neighbor algorithms, dual-alignment pipelines, etc., are capable of facilitating ctDNA-based mutation profiling and prognostic stratification [159, 161]. Furthermore, ML/AI-assisted NGS has been successfully applied for the dynamic quantitative monitoring of low-burden cancers and the detection of minimal residual disease (MRD) [159, 162, 163].

## In vivo stimulation of ctDNA release

Even if the plasma is optimally processed and the molecular genetic analysis is capable of detecting 1 copy of mutated DNA per preparation, small tumors will remain undetected by the LB [47, 125]. Antitumor therapies, particularly local irradiation and tyrosine kinase inhibitors, induce apoptosis of cancer cells and, therefore, render transient spike of ctDNA concentration [24, 51, 96–99, 101, 164–166]. The use of cancer drugs for the support of LB is unlikely to have a practical value: in order to warrant the desirable effect, a given drug should a priori have high antitumor activity. In the real-world setting, LB for visible tumor lumps is performed exactly for the selection of the best drugs, while there is no actual need for ctDNA analysis when the best systemic therapy is already known. For example, lung cancer progression under the treatment by first- or second-generation EGFR inhibitors requires the analysis of *EGFR* T790M mutation, which sometimes can be accomplished by ctDNA testing [139, 144, 167, 168]. Identification of *EGFR* T790M mutation calls for the administration of osimertinib; however, this drug cannot be administered before the test. Irradiation is potentially more practical for the stimulation of ctDNA shedding. Several studies have shown a transient increase in the ctDNA level right after the beginning of radiotherapy [96–99]. However, not all patients demonstrate a ctDNA spike; furthermore, the increase of ctDNA concentration in the bloodstream is generally only within 1.5–2-fold, which may not be sufficient in many clinical situations [51, 169]. All relevant human studies were performed on patients, who received tumor irradiation as a part of the standard treatment plan. It is questionable, whether this intervention can be applied only for the purpose of LB. Furthermore, this approach is potentially feasible only for the analysis of treatment-induced mutations, like *EGFR* T790M, and is not applicable to early cancer detection and monitoring of MRD.

Brain tumors compose a special category of malignancies, as they are separated from the bloodstream by the blood-brain barrier (BBB). Several studies demonstrated that focused ultrasound may facilitate the detection of ctDNA, probably by the disruption of this barrier [50, 52, 100].

## Slowing ctDNA decay

The half-life of ctDNA does not exceed 1–2 hours, with some studies suggesting even shorter estimates [10, 11, 170]. ctDNA undergoes rapid decay. First, cfDNA is efficiently absorbed by liver-resident Kupffer cells and spleen macrophages [40, 42]. In addition, there are circulating nucleases, which accelerate decay of extracellular blood DNA [39, 41]. Martin-Alonso et al. [171] explored these mechanisms of ctDNA clearance in mouse experiments. They injected a succinyl phosphoethanolamine-based liposomal agent to interfere with liver macrophages. In addition, they utilized DNA-binding antibodies in order to protect DNA from nuclease digestion. The combination of these two interventions resulted in a transient increase in the concentration of circulating DNA by more than an order of magnitude [171]. This is theoretically sufficient to allow detection of tumors as small as 1 cm^3^ in diameter. The described approach is highly promising for cancer screening and monitoring of the MRD. Translation of this methodology from mice to humans requires careful consideration of the safety of injected substances.

## Combining ctDNA with other analytes

**Figure 3:**
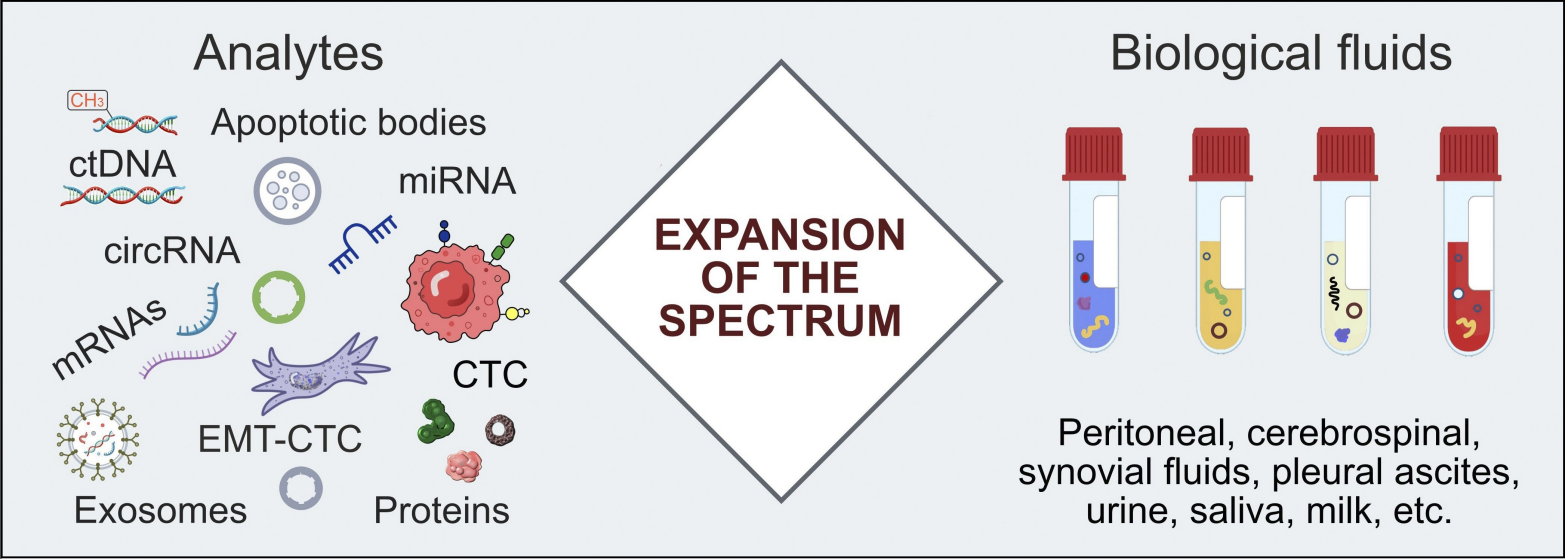
**Beyond plasma ctDNA: the reliability of LB can be increased by expanding the range of analytes and involving biological fluids which are proximal to the tumor site.** circRNA: circular RNA; CTC: circulating tumor cell; ctDNA: circulating tumor DNA; EMT-CTC: circulating tumor cell undergoing epithelial-to-mesenchymal transition; LB: liquid biopsy; miRNA: microRNA; mRNAs: messenger RNAs

While ctDNA can be reliably discriminated from non-tumorous DNA by mutation analysis, other intensively studied analytes, like tumor-derived proteins or cells expressing some cell surface markers, are evidently less cancer-specific. Nevertheless, a simultaneous analysis of several groups of biomarkers, for example, ctDNA, messenger RNA (mRNA), miRNA, CTC, tumor-educated platelets, exosomes, and proteins, may provide some added value, especially in tumor-agnostic screening for early cancers [18, 82, 83, 172–175]. Another underutilized option is to consider non-blood biological fluids that are proximal to the tumor site, such as urine, cerebrospinal fluid, and pleural or peritoneal effusions [18, 176] (Figure 3).

The multianalyte approach has been explored in several blood-based assays, which demonstrated encouraging results in several large-scale investigations [177–180]. The best-known is the so-called CancerSEEK panel, which combines quantitative analysis of eight protein cancer biomarkers and detection of over 1,000 tumor-specific mutations in сtDNA. Initially, the assay was tested on 1,005 patients with non-metastatic, clinically confirmed cancers of the ovary, liver, stomach, pancreas, esophagus, colorectum, lung, or breast [181]. The sensitivity of CancerSEEK has been reported within the range of 69–98% for ovarian, liver, stomach, pancreatic, and esophageal cancers, with only 7 of 812 healthy controls being misrecognized as positive. The subsequent DETECT-A prospective study evaluated the performance of this test in more than 10,000 women aged 65–75 years and showed that the CancerSEEK was able to reveal approximately 1 out of 4 “true” early-stage tumors across the entire spectrum of malignant diseases [7].

Multianalyte assays targeted at the detection of a single cancer type understandably render somewhat better performance. For example, a single-tube analysis of ctDNA, exosomal mRNA, and CA19-9 protein in the blood of pancreatic ductal adenocarcinoma patients demonstrated 92% accuracy, 95% specificity, and 88% sensitivity in revealing cancer disease [182]. Single-organ assays have obvious relevance for the post-surgical follow-up of oncological patients aimed at early detection of tumor relapse, while their potential utility in population-based screening is limited to exceptionally common cancer types and high-risk individuals.

The analysis of ctDNA methylation holds a great promise [183–185]. Tumors differ from normal cells by the pattern of DNA methylation, and these differences are generally more common than mutations [186, 187]. The technologies for the analysis of methylation of ctDNA rely on bisulfite conversion‐based methods [e.g., whole-genome bisulfite sequencing (WGBS), MCTA-seq, ELISA-seq, targeted bisulfite sequencing, etc.] [188–190], enrichment-based approaches combining immunoprecipitation and high-throughput sequencing (SeqCap Epi CpGiant; cfMeDIP‐seq; TET) [191, 192], and various procedures utilizing methyl‐sensitive restriction endonucleases [193, 194]. Several studies demonstrated the utility of methylation analysis of ctDNA for early cancer detection, MRD assessment, estimating treatment response and disease prognosis, etc. [185, 186, 195–200]. Methylation-based techniques are potentially proficient in identifying tumor tissue origin, which is particularly important for cancer screening [186, 199, 201–203].

## Reproducibility of ctDNA assays

Assays that measure ctDNA are influenced by numerous experimental variables and artifacts, many of which remain incompletely understood. Because of the very low concentration in the bloodstream, ctDNA analysis requires PCR amplification and ultra-deep sequencing steps, which may produce various errors. On the other hand, improper blood handling during the collection, transportation, and processing may result in hemolysis and contamination of circulating tumor-derived DNA with genomic DNA. The harmonization of pre-analytical and analytical workflows is essential for the interlaboratory reproducibility of ctDNA-based assays [21, 81, 91, 204].

Several multicenter studies have been conducted to assess the reproducibility of LB results when using different ctDNA extraction methods [205] and various ctDNA detection assays [37, 44, 148]. Circulating mutant fragments, presented with VAF of > 0.5%, were detected with high sensitivity, accuracy, and reproducibility by all participating assays, whereas the detection of VAF at 0.5% or lower proved challenging [44, 206–208]. Meanwhile, ctDNA variants with VAF < 0.5% are particularly important, as they characterize early-stage tumors, MRD, or emerging recurrences. Such cases account for 25–30% of all samples submitted for LB [37, 209]. Here, the most reliable approach to avoid false-negative results is the use of ddPCR for the confirmation and tracking of “tumor-informed” mutations. A combination of synthetic DNA spike-in controls (sequins) [210] and cell-line derived reference samples [211] can be utilized for the monitoring of the analytical performance of ctDNA assays.

The use of standardized commercial kits or centralized services may improve the reproducibility of ctDNA-based LB procedures and enable the comparison of various data sets [212–215]. Several LB kits and diagnostic pipelines have received FDA or EU approval. Therascreen EGFR RGQ Plasma PCR Kit (Qiagen) is a companion test for detection of *EGFR* mutations in lung cancer patients [216]. Guardant360 CDx (Guardant Health) [149] and FoundationOne Liquid CDx (Foundation Medicine/Roche) [217] gene profiling platforms have been designed to support the choice of targeted therapies in multiple tumor types. Epi proColon (Epigenomics) is the first FDA-approved blood-based assay, which relies on the use of methylation DNA markers for colorectal cancer screening [218]. Galleri (Grail) [219] and Signatera (Natera) kits [127] are used in Clinical Laboratory Improvement Amendments (CLIA)-certified labs for MRD detection and multi-cancer screening. Syantra DX^™^ Breast Cancer (Syantra) assay is suggested to support early diagnosis of breast cancer [220].

Surprisingly, the reproducibility of serial ctDNA tests taken from the same patient within a short time interval has not been rigorously assessed yet. Kuligina et al. [70] investigated serial plasma samples from 82 cancer patients, whose tumors contained common hot-spot oncogenic mutations. Moderate physical exercise, recent food, or time of the day did not significantly influence the content of ctDNA. Strikingly, as many as 24/82 (29%) patients showed the presence of mutated ctDNA in some but not all blood draws. Some degree of instability of various laboratory values is well known to clinicians; therefore, many conventional tests are administered repetitively. It needs to be established whether the same attitude is also of benefit in cancer patients.

## Conclusions

ctDNA-based LB is highly important for many areas of clinical oncology, including early cancer detection, prediction of therapeutic response and long-term survival, disease monitoring, and support of various treatment decisions. However, ctDNA analysis is usually performed at the limits of available technologies and, therefore, remains relatively error-prone.

Multiple pre-analytical and analytical factors affect the reproducibility of ctDNA assays. In particular, proper handling of plasma samples as well as the use of ultrasensitive methods for detection of tumor-derived molecules are of paramount significance. Still, the content of ctDNA is usually vanishingly low; therefore, some interventional procedures are currently being assessed. For example, in vivo interference with ctDNA release and decay may lead to a breakthrough in LB performance if proven to be safe. Surprisingly, the reproducibility of ctDNA-based tests has not been rigorously assessed yet; future studies have to put a strong emphasis on this highly important issue.

## Abbreviations

AI: artificial intelligence
ccfDNA: circulating cell-free DNA
cfDNA: cell-free DNA
CTCs: circulating tumor cells
ctDNA: circulating tumor DNA
DBS: dried blood spots
ddPCR: droplet digital PCR
EDTA: ethylenediaminetetraacetic acid
LB: liquid biopsy
miRNAs: microRNAs
ML: machine learning
MRD: minimal residual disease
mRNA: messenger RNA
NGS: next-generation sequencing
UMIs: unique molecular identifiers
VAF: variant allele frequency

## Declarations

### Copyright

© The Author(s) 2025.
